# Clinical management and acute exacerbations in patients with idiopathic pulmonary fibrosis in Spain: results from the OASIS study

**DOI:** 10.1186/s12931-022-02154-y

**Published:** 2022-09-07

**Authors:** Esteban Cano-Jiménez, Ana Dolores Romero Ortiz, Ana Villar, María Jesús Rodríguez-Nieto, Alba Ramon, Silvia Armengol

**Affiliations:** 1 Servicio de Neumología, ILD Unit, Hospital Universitario Lucus Augusti, C/Ulises Romero N 1, 27003 Lugo, Spain; 2 Servicio de Neumología, Hospital Universitario Virgen de Las Nieves, Granada, Spain; 3 Servei de Pneumologia, Hospital Universitari Vall d’Hebron, Barcelona, Spain; 4 Servicio de Neumología, IIS Fundación Jiménez Diaz, CIBERES, Madrid, Spain; 5 Boehringer Ingelheim España, Sant Cugat del Vallès, Spain

**Keywords:** Idiopathic pulmonary fibrosis (IPF), Clinical management, IPF acute exacerbations, IPF management, IPF progression, Early treatment

## Abstract

**Background:**

Idiopathic pulmonary fibrosis (IPF) is a progressive disease associated with decline in lung function and poor prognosis entailing significant impairment in quality of life and high socioeconomic burden. The aim of this study was to characterize clinical management and resources utilization of patients with IPF in Spain, according to predicted forced vital capacity (FVC) % at baseline.

**Methods:**

Prospective, non-interventional, multicentric real-world data study in patients with IPF in Spain with 12-months follow-up. Clinical management and resources utilization during study period were recorded and compared between groups. FVC decline and acute exacerbations occurrence and associated healthcare resource use were also analysed. FVC decline after 12 months was estimated as relative change.

**Results:**

204 consecutive patients with IPF were included and divided according to baseline FVC % predicted value. At baseline, patients with FVC < 50% received significantly more pharmacological and non-pharmacological treatments, and more help from caregiver. During the 12-months follow-up, patients with FVC < 50% required more specialized care visits, emergency visits, hospitalizations, pulmonary functions tests, non-health resource use (special transportation), and pharmacological treatments (p < 0.05 for all comparisons). Moreover, patients with FVC < 50% at baseline experienced more AE-IPF (p < 0.05), requiring more health-related resources use (primary care visits, p < 0.05). FVC decline was observed in all groups over the 12 months. FVC decreased on average by 2.50% (95% CI: − 5.98 to 0.98) along the year. More patients experienced an FVC decline > 10% in the more preserved lung function groups than in the FVC < 50% group, because of their already deteriorated condition.

**Conclusions:**

We observed a significantly higher annual IPF-related resource use in patients with more impaired lung function at baseline. Since FVC decreases irrespective of FVC% predicted at baseline, slowing IPF progression to maintain patients at early disease stages is relevant to improve IPF management and to optimize resource use.

*Trial registration:* EU PAS register number EUPAS19387 [June 01, 2017].

**Supplementary Information:**

The online version contains supplementary material available at 10.1186/s12931-022-02154-y.

## Background

Idiopathic pulmonary fibrosis (IPF) is a chronic, progressive and fatal fibrosing interstitial pneumonia of unknown cause characterized by progressive worsening of dyspnoea and fibrosis and unrecoverable decline in lung function [1, 2]. Data of incidence and prevalence vary depending on the country. In Spain, incidence ranges between 4.6 and 7.4 cases per 100,000 and prevalence is estimated as 13 cases per 100,000 for females and 20 cases per 100,000 for males, resulting in approximately 7500 patients currently affected [3]. Disease progression is variable and difficult to predict, and entails a significant and gradual decline of lung function that reduces patient functioning and quality of life. In addition, some patients may experience acute respiratory exacerbations, which worsen lung function and prognosis [4].

Despite prognosis and mortality of patients with IPF has improved with antifibrotics [5, 6], no new longitudinal studies specifically assessing median survival have been identified. Available data before the antifibrotic era revealed, a median survival of 2.5–3.5 years after IPF diagnosis [7]. Important predictors of mortality are occurrence of acute exacerbations of IPF (AE-IPF) and decline in forced vital capacity (FVC). Having one or more AE-IPF increases the risk of death by tenfold [8], and AE-IPF are associated with a short-term mortality rate of approximately 50% [9, 10]. In addition, a higher annual rate of FVC decline is associated with worse survival: patients with FVC decline > 10% have approximately a twofold increase of risk of death than patients with FVC decline < 5% [8]. Importantly, rates of FVC decline are similar between patients with well-preserved lung function at baseline (FVC > 90% predicted) and patients with less-preserved FVC [11, 12], although patients with less-preserved FVC at baseline are at higher risk of suffering AE-IPF [10].

IPF treatment aims to prevent lung function decline and reduce the occurrence of AE-IPF, in order to improve patient’s quality of life and survival. Absence or delay of treatment might entail a risk in terms of disease progression and patient survival. Nevertheless, and despite the availability of effective approved antifibrotic treatments such as nintedanib and pirfenidone, many patients remain untreated, especially at early stages of the disease [2]. Nintedanib has shown to significantly reduce the decline in lung function [13, 14] and the risk of suffering AE-IPF [14–16]. Treatment with antifibrotics have shown a reduction in mortality in clinical trials [16–18] and, importantly, in real world studies: risk of death was 37% lower in patients receiving antifibrotic therapy than in patients not receiving antifibrotic therapy [5]. Furthermore, patients receiving antifibrotics have a higher median survival after diagnosis (3–3.5 years vs. 2.5 years in untreated patients) [19]. Results from a recent meta-analysis that included around 13,000 patients with IPF from both randomized controlled trials and cohort studies showed that antifibrotics reduce risk of all-cause mortality in 45% [6]. Nintedanib has demonstrated benefit regardless of basal lung function, reducing disease progression by a similar proportion in patients with well-preserved lung function (FVC > 90% predicted) as in patients with worse lung function (FVC < 90% predicted) [12].

Due to its poor prognosis and impact on patients’ lives [20, 21], IPF represents an important socioeconomic burden [22]. In Spain, management of IPF patients has a high economic impact on the Spanish National Health System (NHS), especially for patients with rapid disease progression according to a Delphi panel [23]. In-depth knowledge of current management and real resource use is essential for healthcare providers to optimize resource allocation and reduce the associated costs. Therefore, the aim of this research was to characterize clinical management and resources utilization of patients with IPF in Spain during a follow-up period of 12 months, according to predicted FVC % value at baseline. We also aimed to analyse FVC decline and AE-IPF occurrence, and to estimate the healthcare resource use related to AE-IPF.

## Methods

The OASIS study is a prospective, non-interventional, multicentric real world data study that aimed to characterize clinical management and resources utilization in patients with confirmed IPF in Spain. The study was carried out in 28 secondary care sites throughout Spain. The primary objective of the OASIS study was to estimate the socioeconomic impact of IPF. Key secondary objectives were to define clinical management and resources utilization associated with an AE-IPF event, as well as to characterize the frequency of an AE-IPF event according to FVC % predicted value at baseline and to FVC annual rate of decline. The results of the primary objective of the OASIS study have been reported in a separate publication, currently under peer-review. In this publication, clinical management of IPF and AE-IPF and resources utilization in patients with IPF followed for 12 months according to FVC % predicted value (FVC < 50%, FVC 50–80%, FVC > 80%) at baseline are presented.

### Patient eligibility

Patients were enrolled consecutively from interstitial lung disease (ILD) units of pulmonology services where IPF is diagnosed and managed according to the Spanish health care system. Patients were recruited from December 2017 to July 2018. Inclusion criteria were: confirmed IPF diagnosis according to 2011 ATS/ERS/JRS/ALAT IPF guidelines [1], being ≥ 40 years old, and being able to sign a written informed consent form. Exclusion criteria were: inability to understand Spanish or inability to complete the written informed consent; concomitant participation in any other clinical trial, or inability to conduct the follow-up at the enrolling site.

The study was approved by the Ethical Board (EB) of all participant hospitals. The EB of H. Fundación Jiménez Díaz in Madrid, Spain, acted as reference EB. All patients provided written informed consent prior to their participation.

### Data collection and analysis

Sociodemographic and clinical data were collected from medical records and study questionnaires completed by patients. In order to reduce recall bias, patients were asked to complete a patient diary during the study, which included recording use of IPF-related resources (health and non-health related). Follow-up was performed during one year, and data was collected at 3 visits, as per clinical practice: the baseline visit and the closest visits to 6 and 12 months from baseline. Data for AE-IPF were reported independently to the visits. In this study, AE-IPF was defined as an acute, clinically significant respiratory deterioration characterized by evidence of new widespread alveolar abnormality [24]. AE-IPF and its management were registered in each visit. Each investigator checked the data from patient diary and medical records and addressed inconsistencies with the patients during the visits. Reconciled data was entered in the electronic case report form (eCRF).

Resources evaluated in this study included only those IPF-related: primary and secondary care visits, outpatient visits, emergency visits (primary care and hospital), hospitalizations, intensive care unit (ICU) with and without intubation, outpatient tests and other examinations, use of transport, use of formal and informal caregiver, pharmacological and non-pharmacological treatments related to IPF, orthopaedic material, formal social services, economic aid and structural adaptations, and days off work.

FVC decline was estimated as relative change as follows: [(Final FVC % predicted – Initial FVC % predicted) / Initial FVC % predicted] × 100.

### Statistical analysis

A descriptive analysis was performed of all the variables recorded for the study population. For continuous quantitative variables, the mean, standard deviation (SD), and valid n were calculated. Categorical variables were presented as absolute and relative frequencies (percentages).

For bivariate analysis, continuous variables were compared across subgroups of population using two-sample t-tests or analysis of variance (ANOVA) or the Mann–Whitney U test or Kruskal–Wallis test, as appropriate. The categorical variables were analysed using the Chi-square or Fisher test, as appropriate. A statistical significance level of 0.05 was applied in all the statistical tests. The evaluation was carried out using SAS® software, version 9.4.

## Results

**Fig. 1:**
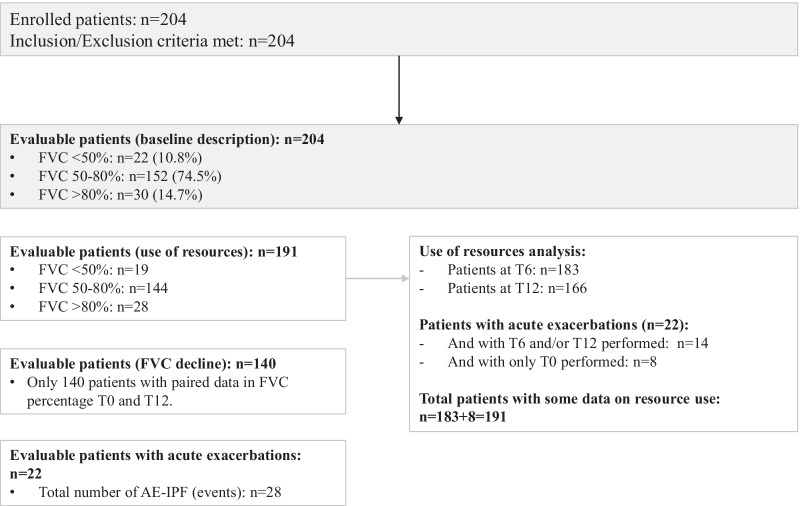
Flowchart of participants. *AE-IPF* acute exacerbation of IPF, *FVC* forced vital capacity, *IPF* idiopathic pulmonary fibrosis

A total of 204 consecutive patients with IPF met the selection criteria and were enrolled in the study. Patients were divided according to FVC % predicted value at baseline: 22 (10.8%) patients had a FVC < 50%, 152 (74.5%) a FVC 50–80% and 30 (14.7%) a FVC > 80%. Final evaluable population with data on resource use included 191 subjects, with a mean (SD) follow-up of 12.40 (1.07) months (Fig. 1).

### Baseline sociodemographic and clinical characteristics of the study population

Overall, 77.0% of the patients were males, and mean (SD) age was 70.80 (7.60) years. The group with more preserved lung function at baseline (FVC > 80%) had a higher percentage of active workers (p = 0.0232) (Table 1). No significant differences between groups were observed in BMI, smoking habit, and exposure to occupational and/or environmental risk factors. Of note, although all patients had been diagnosed with IPF, 47.5% of them had an environment and/or occupational exposure (Table 1).

**Table 1:** Baseline characteristics of the study population by FVC% predicted at baseline

Characteristic	Total sample N = 204	Predicted FVC% at baseline			
		FVC < 50% N = 22	FVC 50–80% N = 152	FVC > 80% N = 30	p value
Sex, male, n (%)	157 (77.0%)	18 (81.8%)	120 (78.9%)	19 (63.3%)	0.1516
Age (years), mean (SD)	70.80 (7.60)	70.32 (8.52)	71.36 (7.21)	68.33 (8.54)	0.1992
Employment status (active workers), n (%)	24 (11.8%)	2 (9.1%)	14 (9.2%)	8 (26.7%)	**0.0232**
BMI (kg/m^2^), mean (SD)^†^	28.13 (3.97)	27.15 (3.73)	28.29 (3.88)	28.06 (4.62)	0.5682
Occupational and/or environmental exposure to risk factors, n (%)	97 (47.5%)	10 (45.5%)	75 (49.4%)	12 (40.0%)	0.7718
Smoking habit, n (%)					0.5300
Non smokers	64 (31.4%)	8 (36.4%)	48 (31.6%)	8 (26.7%)	
Former smokers^a^	135 (66.2%)	14 (63.6%)	101 (66.4%)	20 (66.7%)	
Smokers	5 (2.4%)	0 (0.0%)	3 (2.0%)	2 (6.6%)	
Time since IPF diagnosis to baseline visit (years), mean (SD)	1.92 (1.85)	2.00 (1.69)	1.95 (1.91)	1.73 (1.67)	0.6416
Lung function, mean (SD)					
FVC % predicted	65.78 (14.42)	41.96 (5.83)	64.66 (8.42)	88.94 (8.35)	** < 0.0001**
FVC annual rate of decline (%)	5.12 (5.84)	8.40 (6.24)	4.68 (5.70)	4.50 (5.83)	0.1055
DL_co_-c % predicted^†^	49.99 (17.39)	36.17 (12.27)	50.29 (17.36)	57.83 (15.29)	** < 0.0001**
Six-minute walk test, mean (SD)^†^					
Distance (m), mean (SD)	443.70 (101.32)	376.45 (122.65)	449.78 (92.71)	472.55 (103.70)	**0.0036**
Need for oxygen, n (%)	17 (10.7%)	5 (25.0%)	12 (10.3%)	0 (0.0%)	**0.0307**
Comorbidities associated with IPF, n (%)^b^	147 (72.1%)	18 (81.8%)	110 (72.4%)	19 (63.3%)	0.3358
Arterial hypertension	71 (48.3%)	7 (38.9%)	58 (52.7%)	6 (31.6%)	0.1629
Diabetes	35 (23.8%)	4 (22.2%)	26 (23.6%)	5 (26.3%)	0.9482
Gastroesophageal reflux	32 (21.8%)	2 (11.1%)	27 (24.5%)	3 (15.8%)	0.4268
Coronary heart disease	21 (14.3%)	4 (22.2%)	16 (14.5%)	1 (5.3%)	0.3447
Sleep apnoea-hypopnea syndrome	19 (12.9%)	3 (16.7%)	15 (13.6%)	1 (5.3%)	0.5468

In bold, p-values < 0.05

*BMI* body mass index, *DL*_*CO*_*-c* carbon monoxide lung diffusion capacity (corrected for haemoglobin), *FVC* forced vital capacity, *IPF* idiopathic pulmonary fibrosis, *SD* standard deviation

^†^There were missing values

^a^Former smoker: person who, having smoked, has maintained abstinence for at least the last 6 months

^b^Shown are the comorbidities suffered by at least 10% of patients

Significant differences in clinical characteristics were observed between groups with different FVC % predicted at baseline: predicted corrected diffusing capacity for carbon monoxide (DLCO-c) was higher in patients with more preserved FVC than in patients with lower FVC % predicted at baseline (p < 0.0001). As expected, patients with lower FVC % predicted at baseline also achieved significantly shorter distances in the 6-min walk test (6MWT) than patients with more preserved FVC (p = 0.0036) (Table 1). Lastly, 72.1% of the patients had some comorbidity associated with IPF at baseline, with no differences between groups. The most common comorbidities were arterial hypertension (48.3%), diabetes (23.8%), and gastroesophageal reflux (21.8%) (Table 1).

Regarding management of IPF at baseline, antifibrotic therapy was the most prescribed treatment, with 81.4% of patients receiving antifibrotics, followed by antacids (35.3% of participants) and non-pharmacological treatment (20.1% of participants, mainly oxygen therapies). It should be noted that up to 30% of patients in the group with more preserved FVC (FVC > 80%) did not receive antifibrotics (Table 2).

**Table 2:** Management of IPF patients at baseline, according to predicted FVC%

Management intervention	Total sample N = 204	Predicted FVC% at baseline			
		FVC < 50% N = 22	FVC 50–80% N = 152	FVC > 80% N = 30	p value
Patients receiving a pharmacological treatment associated with IPF, n (%)^a^					
Antifibrotic^b^	166 (81.4%)	16 (72.7%)	129 (84.9%)	21 (70.0%)	0.0876
Systemic corticosteroids^c^	10 (4.9%)	4 (18.2%)	5 (3.3%)	1 (3.3%)	**0.0320**
Antiacids^d^	72 (35.3%)	7 (31.8%)	57 (37.5%)	8 (26.7%)	0.4921
Antibiotics for systemic use^e^	6 (2.9%)	1 (4.5%)	4 (2.6%)	1 (3.3%)	0.6290
Other^f^	11 (5.4%)	2 (9.1%)	7 (4.6%)	2 (6.7%)	-
Patients receiving a non-pharmacological treatment associated with IPF, n (%)^a^					
Total, n (%)	41 (20.1%)	14 (63.6%)	26 (17.1%)	1 (3.3%)	** < 0.0001**
Liquid oxygen therapy, n (%)	13 (6.4%)	5 (22.7%)	7 (4.6%)	1 (3.3%)	**0.0142**
Electric portable oxygen therapy, n (%)	9 (4.4%)	2 (9.1%)	7 (4.6%)	0 (0.0%)	0.2849
Oxygen therapy concentrator, n (%)	12 (5.9%)	6 (27.3%)	6 (3.9%)	0 (0.0%)	**0.0007**
Oxygen therapy portable device, n (%)	6 (2.9%)	2 (9.1%)	3 (2.0%)	1 (3.3%)	0.1153
Non-invasive mechanical ventilation, n (%)	3 (1.5%)	2 (9.1%)	1 (0.7%)	0 (0.0%)	**0.0410**
High Flow Nasal Cannulas (HFNC), n (%)	0 (0.0%)	0 (0.0%)	0 (0.0%)	0 (0.0%)	**–**
Flu and pneumococcal vaccination, n (%)	9 (4.4%)	4 (18.2%)	5 (3.3%)	0 (0.0%)	**0.0153**
Nutritional supplements, n (%)	2 (1.0%)	2 (9.1%)	0 (0.0%)	0 (0.0%)	**0.0112**
Other, n (%)	1 (0.5%)	1 (4.5%)	0 (0.0%)	0 (0.0%)	0.1078
Patients receiving help from a caregiver, n (%)	41 (20.1%)	9 (40.9%)	27 (17.8%)	5 (16.7%)	**0.0356**
Formal, n (%^‡^)	2 (4.9%)	1 (11.1%)	0 (0.0%)	1 (20.0%)	0.1110
Informal, n (%^‡^)	39 (95.1%)	8 (88.9%)	27 (100.0%)	4 (80.0%)	

In bold, p-values < 0.05

*FVC* forced vital capacity, *IPF* idiopathic pulmonary fibrosis, *SD* standard deviation

^a^Either to treat IPF or to treat some comorbidity or symptomatology associated with IPF. Did not include the treatments administered during a hospitalization

^b^Includes nintedanib (44.6% of all patients receiving antifibrotics [74/166]) and pirfenidone (55.4% [92/166])

^c^Includes methylprednisolone and prednisone

^d^Includes esomeprazole, lansoprazole, omeprazole, pantoprazole, rabeprazole and ranitidine

^e^Includes azithromycin and sulfamethoxazole plus trimethoprim

^f^Includes: anticoagulants (dabigatran etexilate), anti-HTAP (Phosphodiesterase-5 Blockers) (sildenafil), diuretics (furosemide), calcium. combinations with vitamin D and/or other drugs, immunosuppressants, alendronic acid, amlodipine, amphotericin B, calcifediol, calcium carbonate, folic acid, furosemide, indacaterol and glycopyrronium bromide, ipratropium bromide, tiotropium bromide, umeclidinium bromide, vilanterol and fluticasone furoate

^‡^Over total number of patients receiving help from a caregiver in the total sample (n=41) and in each FVC% predicted group

Several differences in IPF management were observed between groups: patients with FVC% predicted at baseline < 50% received significantly more systemic corticosteroids, more non-pharmacological treatments (ventilatory support, nutritional supplements and the flu/pneumococcal vaccine) and more help from a caregiver than patients with more preserved FVC% predicted at baseline (Table 2).

### Health and non-health related resources use at 12 months

During the study, 30.9% of patients attended primary care visits, almost all patients (97.9%) attended specialized care visits (pulmonologist) and 24.1% required emergency visits (Table 3). Patients with FVC < 50% predicted at baseline required a mean of 2.47 primary care visits, vs. 0.86 and 0.50 in the FVC 50–80% and FVC > 80% predicted groups, respectively (p = 0.0570) (Table 3). Similarly, patients with FVC < 50% predicted at baseline required a mean of 4.16 pulmonologist visits, vs. 3.28 and 2.93 in the FVC 50–80% and FVC > 80% predicted groups, respectively (p = 0.2758, data not shown). Emergency room visits were significantly more common among patients with less preserved FVC at baseline, with 57.9% of patients with FVC < 50% predicted at baseline vs. 21.5% and 14.3% for those in the FVC 50–80% and FVC > 80% predicted groups (p = 0.0412) (Table 3).

**Table 3:** Health resource used for IPF management (including AE-IPF) during follow-up, according to baseline predicted FVC%

Health resource	Total N = 191	FVC < 50% N = 19	FVC 50–80% N = 144	FVC > 80% N = 28	P value
*Visits*					
Patients who required primary care visits, n (%)	59 (30.9%)	9 (47.4%)	43 (29.9%)	7 (25.0%)	0.2296
Number of visits to primary care, mean (SD)	0.97 (2.13)	2.47 (3.29)	0.86 (2.03)	0.50 (1.00)	0.0570
Patients who required specialized care visits, n (%)	187 (97.9%)	17 (89.5%)	143 (99.3%)	27 (96.4%)	**0.0275**
Pulmonologist	187 (97.9%)	17 (89.5%)	143 (99.3%)	27 (96.4%)	**0.0275**
Nurse	59 (30.9%)	4 (21.1%)	42 (29.2%)	13 (46.4%)	0.1207
Nutritionist	5 (2.6%)	2 (10.5%)	2 (1.4%)	1 (3.6%)	0.0598
Psychiatrist	7 (3.7%)	1 (5.3%)	5 (3.5%)	1 (3.6%)	0.8105
Psychologist	3 (1.6%)	0 (0.0%)	2 (1.4%)	1 (3.6%)	0.5737
Other health professional^a^	37 (19.4%)	5 (26.3%)	26 (18.1%)	6 (21.4%)	0.6629
Respiratory rehabilitation	16 (8.4%)	4 (21.1%)	9 (6.3%)	3 (10.7%)	0.0611
Nursing home visit	3 (1.6%)	1 (5.3%)	1 (0.7%)	1 (3.6%)	0.1504
Smoking consult cessation	1 (0.5%)	0 (0.0%)	1 (0.7%)	0 (0.0%)	1.0000
Patients who required emergency visits, n (%)	46 (24.1%)	11 (57.9%)	31 (21.5%)	4 (14.3%)	**0.0412**
Primary care area^b^	15 (32.6%)	4 (36.4%)	10 (32.3%)	1 (25.0%)	**0.0055**
Hospital care area^b^	38 (82.6%)	8 (72.7%)	27 (87.1%)	3 (75.0%)	1.0000
*Hospitalizations*					
Patients who required a hospital admission due to IPF, n (%)^c^	29 (15.2%)	9 (47.4%)	17 (11.8%)	3 (10.7%)	**0.0010**
Number of hospitalizations/patient, mean (SD)	0.34 (0.96)	0.89 (1.24)	0.28 (0.93)	0.29 (0.76)	**0.0010**
Days of hospitalization, mean (SD)	8.25 (7.23)	13.53 (10.01)	6.26 (4.73)	5.71 (3.04)	**0.0059**
Patients who were admitted to ICU, n (%)^d^	6 (11.3%)	3 (20.0%)	3 (9.7%)	0 (0.0%)	0.4573
Days in ICU, mean (SD)	12.17 (8.57)	10.00 (8.66)	14.33 (9.71)	–	0.3758
Patients who required intubation during ICU admission, n (%)^e^	5 (83.3%)	3 (100.0%)	2 (66.7%)	–	1.0000
*Tests*					
Patients who required laboratory tests, n (%)^f^	167 (87.4%)	18 (94.7%)	127 (88.2%)	22 (78.6%)	0.2231
Patients who required pulmonary function tests, n (%)^g^	161 (84.3%)	12 (63.2%)	127 (88.2%)	22 (78.6%)	**0.0125**
Other tests, n (%)^h^	88 (46.1%)	7 (36.8%)	65 (45.1%)	16 (57.1%)	0.3529

In bold, p-values < 0.05

*FVC* forced vital capacity, *ICU* intensive care unit, *IPF* idiopathic pulmonary fibrosis, *NIMV* non-invasive mechanical ventilation, *SD* standard deviation

^a^Includes: allergist, anesthesiologist, cardiologist, surgeon, dermatologist, digestive, endocrine, hospital pharmacy/pharmacy, physiotherapist, hematologist, internist, nephrologist, neurologist, preventive/preventive medicine, radiologist, rheumatologist, urologist

^b^Over total number of patients who required emergency visits in the total sample (n=46), and in each FVC% predicted group

^c^A total of 29 patients required 53 hospital admissions due to IPF

^d^Over total number hospital admissions in each group

^e^Over total number ICU admissions in each group

^f^Included: hemogram, biochemistry, coagulation profile, erythrocyte sedimentation rate, liver profile, angiotensin converting enzyme, rheumatoid factor, antinuclear antibodies, C-reactive protein, procalcitonin, natriuretic peptide, dimer D and “another test”

^g^Included: spirometry, pulmonary plethysmography, carbon monoxide diffusion capacity, 6-min walk test, and “another test”

^h^Included: X-Ray, High resolution computed tomography (HRCT), bronchoscopy, bronchoalveolar lavage, transbronchial biopsy, arterial blood gases, PCR, and “another test”

Regarding hospital admissions, a total of 29 patients required 53 hospital admissions due to IPF. Hospitalizations were more frequent (p = 0.0010) and hospital stay was significantly longer (p = 0.0059) in patients with FVC < 50% predicted at baseline than in patients with more preserved FVC. Among the 53 hospital admissions, 11.3% required ICU admission, but no patient from the baseline FVC > 80% predicted group (Table 3).

Laboratory tests were performed to 87.4% of patients without differences between groups (Table 3). In contrast, pulmonary function tests were significantly different between groups (63.2%, 88.2% and 78.6% patients with FVC < 50%, FVC 50–80%, and FVC > 80%, respectively; p = 0.0125) (Table 3).

During the study period, 344 pharmacological treatments were used (including those newly prescribed for AE-IPF) (Table 4). The most prescribed pharmacological treatment was antifibrotic therapy, representing 58.4% of the total. Specifically, nintedanib represented 49.3% and pirfenidone 50.7% of the prescribed antifibrotics. Prescription pattern of pharmacological treatments was different between groups with different lung function at baseline. Antifibrotics were the most common treatment used among patients with less impaired lung function at baseline, representing 69.2% and 63.5% of the prescribed treatments in FVC > 80% and FVC 50–80% groups, respectively vs. 26.4% in the FVC < 50% group. Antiacids represented 18.9% of the prescribed treatments (Table 4). Regarding non-pharmacological treatments, most common prescriptions were oxygen therapies and flu and pneumococcal vaccination (16.2%), and no difference in prescription pattern was observed between groups (Table 4).

**Table 4:** Prescribed treatments for IPF management (including comorbidities) at 12 months according to baseline predicted FVC%

Prescribed treatments	Total	FVC < 50%	FVC 50–80%	FVC > 80%	P value
**Total number of pharmacological** **treatments, n (%)**^†^	**344 (100.0%)**	**53 (100.0%)**	**252 (100.0%)**	**39 (100.0%)**	** < 0.0001**
Antifibrotic^a^	201 (58.4%)	14 (26.4%)	160 (63.5%)	27 (69.2%)	
Antacids^b^	65 (18.9%)	5 (9.4%)	54 (21.4%)	6 (15.4%)	
Systemic corticosteroids^c^	23 (6.7%)	8 (15.1%)	15 (6.0%)	0 (0.0%)	
Antibiotics for systemic use^d^	15 (4.4%)	9 (17.0%)	4 (1.6%)	2 (5.1%)	
Mucolytics^e^	2 (0.6%)	0 (0.0%)	2 (0.8%)	0 (0.0%)	
Anticoagulants^f^	2 (0.6%)	1 (1.9%)	1 (0.4%)	0 (0.0%)	
Other^g^	36 (10.5%)	16 (30.2%)	16 (6.3%)	4 (10.3%)	
**Total number of non-pharmacological treatments, n (%)**^‡^	**74 (100.0%)**	**27 (100.0%)**	**43 (100.0%)**	**4 (100.0%)**	0.6688
Liquid oxygen therapy	13 (17.6%)	5 (18.5%)	7 (16.3%)	1 (25.0%)	
Electric portable oxygen therapy	14 (18.9%)	5 (18.5%)	9 (20.9%)	0 (0.0%)	
Oxygen therapy with oxygen concentrator	16 (21.6%)	6 (22.2%)	9 (20.9%)	1 (25.0%)	
Oxygen therapy portable device	12 (16.2%)	2 (7.4%)	8 (18.6%)	2 (50.0%)	
NIMV	3 (4.1%)	2 (7.4%)	1 (2.3%)	0 (0.0%)	
Flu and pneumococcal vaccination	12 (16.2%)	4 (14.8%)	8 (18.6%)	0 (0.0%)	
Nutritional supplements	2 (2.7%)	2 (7.4%)	0 (0.0%)	0 (0.0%)	
Other^h^	2 (2.7%)	1 (3.7%)	1 (2.3%)	0 (0.0%)	

*FVC* forced vital capacity, *ICU* intensive care unit, *IPF* idiopathic pulmonary fibrosis, *NIMV* non-invasive mechanical ventilation, *SD* standard deviation

^a^Includes nintedanib (99 prescriptions, 49.3% of all antifibrotic treatments) and pirfenidone (102 prescriptions, 50.7% of all antifibrotic treatments)

^b^Includes esomeprazole, lansoprazole, omeprazole, pantoprazole, rabeprazole and ranitidine

^c^Includes methylprednisolone and prednisone

^d^Includes azithromycin, levofloxacin, sulfamethoxazole plus trimethoprim, cefditoren, amoxicillin/clavulanic acid, ceftazidime, cefuroxime, and colistimethate sodium

^e^Includes acetylcysteine and carbocisteine

^f^Includes dabigatran etexilate and enoxaparin

^g^Includes antidiarrheal. anti-inflammatory /anti-infective intestinal agents, calcium. combinations with vitamin d and/or other drugs, immunosuppressants, alendronic acid, amlodipine, amphotericin B, bisoprolol, calcium carbonate, dexchlorpheniramine, dextromethorphan, fentanyl, folic acid, furosemide, indacaterol and glycopyrronium bromide, ipratropium bromide, isoniazide, loperamide in combination, metoclopramide, morphine, mycophenolic acid, tacrolimus, valganciclovir, among others

^h^Includes physiotherapy and other treatments

^†^Over total number of pharmacological treatments prescribed in the total sample (n=344) and in each FVC% predicted group

^‡^Over total number of non-pharmacological treatments prescribed in the total sample (n=74) and in each FVC% predicted group

Regarding non-health related resources, patients with FVC < 50% used special means of transport, such as ambulance or taxi, to go to the hospital more than other groups (p = 0.0021). Transport was required by 11.0% of patients: 90.5% used the ambulance and 14.3%, taxi. Only 6 patients (3.6%) required the use of orthoprosthetic material. Overall, 22.4% of the patients needed any help from a caregiver along the study, mostly informal caregiver (95.1%) with a mean (SD) of 45.05 (51.48) hours/week dedicated to the patient care. During the study period, no statistically significant differences between FVC predicted were observed regarding the need for a caregiver (Additional file 1: Table S1).

### Acute exacerbations at 12 months according to FVC% predicted at baseline

Along the study, 22 (10.8%) patients experienced a total of 28 exacerbations. Of the 30 patients who died along the study, 14 (46.7%) had suffered at least 1 AE-IPF at some point during the study. In the overall population, the mean (SD) AE-IPF per patient was 0.14 (0.44), and differences in the incidence of AE-IPF between baseline FVC % predicted groups were observed (Table 5). A higher proportion of patients suffered an AE-IPF in the baseline FVC < 50% predicted group (27.3%) than in the groups with more preserved FVC at baseline (10.0% and 8.6% in FVC > 80% and FVC 50–80% groups, respectively) (p = 0.0333) (Table 5).

**Table 5:** Acute exacerbations at 12 months according to predicted FVC% at baseline

	Total N = 204	FVC < 50% N = 22	FVC 50–80% N = 152	FVC > 80% N = 30	p-value
Patients who experienced an AE-IPF during the study, n (%)	22 (10.8%)	6 (27.3%)	13 (8.6%)	3 (10.0%)	**0.0333**
Number of AE-IPF per patient^a^, mean (SD)	0.14 (0.44)	0.41 (0.80)	0.11 (0.38)	0.10 (0.31)	**0.0255**
Number of exacerbations by patient—group^a^, n (%)					0.0540
0 exacerbations	182 (89.2%)	16 (72.7%)	139 (91.4%)	27 (90.0%)	
1 exacerbation	18 (8.8%)	4 (18.2%)	11 (7.2%)	3 (10.0%)	
> 1 exacerbations	4 (2.0%)	2 (9.1%)	2 (1.3%)	0 (0.0%)	
Number of AE-IPF per patient with AE^b^ , mean (SD)	1.27 (0.63)	1.50 (0.84)	1.23 (0.60)	1.00 (0.00)	0.4562
Duration of AE-IPF (in days), mean (SD)	16.50 (18.38)	12.67 (6.32)	20.56 (23.15)	6.33 (5.03)	0.2259

In bold, p-values < 0.05

22 patients reported 28 AE-IPF events through the study period. *AE-IPF* acute exacerbation of IPF, *FVC* forced vital capacity, *IPF* idiopathic pulmonary fibrosis, *SD* standard deviation

^a^Over all patients (n = 204). Patient without exacerbation was imputed 0 exacerbations

^b^Over the patients with exacerbations (n = 22)

### Health and non-health related resources use for acute exacerbations

Overall, 28.6% of AE-IPF episodes required primary care visits and 46.4% specialized care visits: patients visited mainly to pulmonologist (100.0%) and nurse (23.1%) (data not shown), with no statistically significant differences between predicted FVC % groups observed. Overall, 75% of the AE-IPF required emergency room visits, mainly to the hospital care area (95.2%), with significant differences on primary care area visits between groups (p = 0.0055) (Table 6).

**Table 6:** Health-related resources used for AE-IPF events along the study according to predicted FVC% at baseline

	Total N = 28	FVC < 50% N = 9	FVC 50–80% N = 16	FVC > 80% N = 3	p-value
*Visits*					
Primary care visits due to AE-IPF, n (%)	8 (28.6%)	4 (44.4%)	2 (12.5%)	2 (66.7%)	0.0760
Specialized care visits due to AE-IPF, n (%)	13 (46.4%)	4 (44.4%)	8 (50.0%)	1 (33.3%)	1.0000
Emergency visits related to AE-IPF, n (%)	21 (75.0%)	6 (66.7%)	14 (87.5%)	1 (33.3%)	0.1019
Primary care area^a^	6 (28.6%)	4 (66.7%)	1 (7.1%)	1 (100.0%)	**0.0055**
Hospital care area^a^	20 (95.2%)	6 (100.0%)	13 (92.9%)	1 (100.0%)	1.0000
*Hospitalizations*					
Hospital admissions due to AE-IPF, n (%)	21 (75.0%)	8 (88.9%)	11 (68.8%)	2 (66.7%)	0.4529
Number of hospitalizations/event, mean (SD)	0.79 (0.50)	0.89 (0.33)	0.69 (0.48)	1.00 (1.00)	0.5133
Days of hospitalization, mean (SD)	8.48 (5.90)	10.50 (5.48)	8.17 (6.31)	4.33 (4.04)	0.2856
ICU admission, n (%)^b^	2 (8.7%)	0 (0.0%)	2 (16.7%)	0 (0.0%)	0.6206
Days in ICU, mean (SD)	18.50 (9.19)	–	18.50 (9.19)	–	–
Need for intubation, n (%)	2 (100.0%)	0 (0.0%)	2 (100.0%)	0 (0.0%)	–
*Tests*					
Laboratory tests, n (%)^c^	16 (57.1%)	6 (66.7%)	8 (50.0%)	2 (66.7%)	0.8579
Pulmonary function tests, n (%)^d^	2 (7.1%)	0 (0.0%)	2 (12.5%)	0 (0.0%)	0.6190
Other tests, n (%)^e^	16 (57.1%)	6 (66.7%)	8 (50.0%)	2 (66.7%)	0.8579
*Treatments*					
Pharmacological treatments for AE-IPF administered, n (%)	14 (50.0%)	5 (55.6%)	7 (43.8%)	2 (66.7%)	0.7575
Non-pharmacological treatments for AE-IPF administered, n (%)	6 (21.4%)	1 (11.1%)	5 (31.3%)	0 (0.0%)	0.4103

In bold, p-values < 0.05

*AE-IPF* acute exacerbation of IPF, *FVC* forced vital capacity, *ICU* intensive care unit, *IPF* idiopathic pulmonary fibrosis, *SD* standard deviation

^a^Over 21 emergency visits

^b^Over 23 admissions

^c^Included: hemogram, biochemistry, coagulation profile, erythrocyte sedimentation rate, liver profile, C-reactive protein, procalcitonin, natriuretic peptide, dimer D and urine culture

^d^Included: spirometry, carbon monoxide diffusion capacity, and 6-min walk test

^e^Included: X-Ray, High resolution computed tomography (HRCT), arterial blood gases, respiratory virus screening, echocardiogram, and blood culture

A total of 21 patients required 23 hospital admissions due to AE-IPF, representing 75% of all AE-IPF events. Among the AE-IPF events that required hospitalization, 8.7% were admitted in the ICU. Up to 88.9% of patients with FVC < 50% required hospital admissions due to AE-IPF vs. 68.8% and 66.7% in FVC 50–80% and FVC > 80% groups, respectively, although no statistically significant differences between predicted FVC% groups were observed.

Laboratory tests were performed in 57.1% of AE-IPF, pulmonary function tests were performed in 7.1% of the AE-IPF events, and other tests in 57.1% of AE-IPF, without differences between predicted FVC % groups (Table 6).

During the study, 14 of the 28 exacerbation events (50%) required specific pharmacological treatments due to AE-IPF (n = 17 prescriptions). Among patients with AE-IPF, they received pharmacological treatment due to AE-IPF in 50% of AE-IPF episodes (32.1% systemic corticosteroids and 7.1% antibiotics for systemic use, data not shown), without differences between groups (Table 6). Overall, 21.4% of AE-IPF events received a non-pharmacological treatment (different types of supplementary oxygen therapy) (Table 6).

In total, transport related to AE-IPF was required in 42.9% of the AE-IPF events: all (100%) required the ambulance. The events occurring in patients with FVC < 50% and FVC 50–80% were the ones that needed the transport (44.4% and 50% respectively). Use of orthopaedic material or need for structural changes related to an AE-IPF was anecdotal. Only 1 event required orthopaedic material and another one required to make structural changes at home. None of the patients experiencing an AE-IFP received economic aid or formal services neither required a caregiver due to the AE-IPF episode (Additional file 1: Table S2).

### FVC decline at 12 months according to FVC% predicted at baseline

**Fig. 2:**
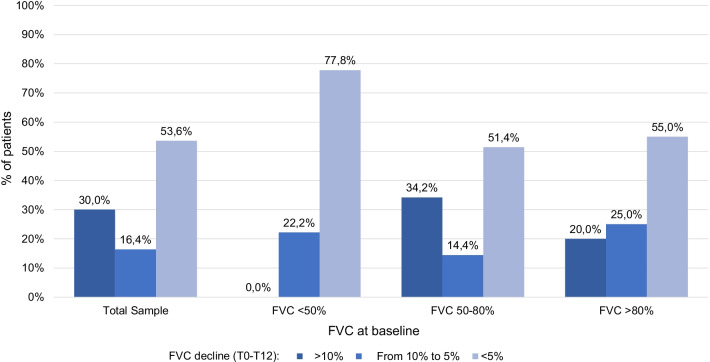
FVC decline at 12 months according to FVC% predicted values at baseline. FVC: forced vital capacity; FVC DECLINE as Relative change: [(Final FVC % predicted – Initial FVC % predicted) / Initial FVC % predicted] × 100

FVC decline (relative change of FVC % predicted value at 12 months vs. baseline) was estimated. FVC decreased on average by 2.50% (95% CI: − 5.98 to 0.98) along the year. No significant differences on the rate of FVC decline between groups (p = 0.1131) were observed (Fig. 2). Of note, more patients experienced an FVC decline >10% in the baseline FVC 50-80% and FVC >80% predicted groups (34.2% and 20.0% of patients, respectively) than in the baseline FVC <50%, were no patient showed FVC declines >10% (Fig. 2), probably because their pulmonary function was already severely impaired.

### FVC decline at 12 months according to acute exacerbations

**Fig. 3:**
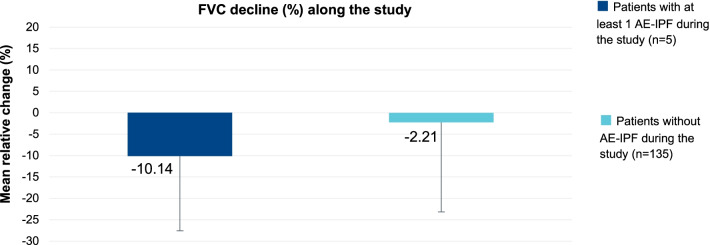
FVC decline at 12 months in patients with or without acute exacerbation. FVC: forced vital capacity; FVC DECLINE as Relative change: [(Final FVC % predicted – Initial FVC % predicted) / Initial FVC % predicted] × 100. Error bars describe standard deviation

FVC decline at 12 months was calculated for patients who had or not experienced an AE-IPF. Patients who had suffered an AE-IPF had a mean FVC decline rate of − 10.14% (17.41) [mean% (SD)] in comparison to a mean decline of − 2.21% (20.95) among those who had not experienced an AE-IPF. This difference was not statistically significant (p = 0.4385) no matter the large numerical difference, probably owing to the limited sample size of exacerbations (Fig. 3).

## Discussion

To the best of our knowledge, this is the first real-world study characterizing health and non-health related resource use and clinical management of IPF patients in Spain during 12 months according to FVC% predicted at baseline.

The main demographic and clinical characteristics of IPF patients included in this study are consistent with those previously described in the literature by the IPF National Registry of the Spanish Respiratory Society (SEPAR) [25], and also in line with other international IPF registries [26–28]. Of note, 47.5% of the patients from this study were associated with environmental exposure to risk factors for pulmonary fibrosis. This may be due to the fact that in daily clinical practice no exhaustive anamnesis of risk factors/environmental exposure is performed and so the diagnosis remains as IPF. Nevertheless, all participants complied with international diagnosis criteria for IPF [1].

IPF is associated with higher healthcare resource use and has a significant economic impact on healthcare systems; however, studies that detail healthcare resource use in IPF and analyse the effect of disease stage and/or progression on healthcare resource use are scarce [22, 23, 27, 29, 30]. Our study shows that patients with less preserved lung function at baseline have a greater healthcare resource use than patients with more preserved lung function. Emergency visits and hospitalizations were significantly higher in patients with FVC < 50% predicted at baseline. This is in agreement with a recent study on hospital-related resource use and costs in a US prospective registry of patients with IPF [29]. Fan et al. reported an overall probability of hospitalization of 30.2% at 12 months that increased up to 51% among those patients with FVC < 50% predicted at baseline [29]. Similarly, a Delphi study carried out in Spain reported that economic impact of IPF was higher in those patients with rapid disease progression [23]. Similarly, higher healthcare resource use and costs have been associated to AE-IPF in other studies [22, 31].

Antifibrotic therapy (nintedanib and pirfenidone) slows disease progression by reducing the rate of FVC decline, and nintedanib, in particular, has demonstrated a reduction of the incidence of AE-IPF [6, 11, 13, 16] and is recommended by international guidelines for the treatment of IPF [32]. Moreover, recent data indicate that antifibrotic treatment significantly improves patient survival [5, 19, 33]. Nevertheless, antifibrotics are not always prescribed in clinical practice, and several studies and patient registries have reported that roughly 60 to 70% of patients with IPF receive antifibrotic treatment [2, 25, 27, 28, 34, 35]. Although use of antifibrotics may vary between international registries and it is difficult to compare due to temporal and geographical drug access differences, low prescription to patients with preserved lung function and/or a “mild” or stable disease has been consistently reported [2, 25, 27, 28, 34, 35]. Data from our study shows an overall higher percentage of antifibrotic prescription (81.4% of all patients), but confirms that physicians tend to undertreat those patients with preserved lung function, as 30% of patients with FVC > 80% predicted at baseline were not receiving antifibrotics.

Progressive decline of FVC was observed in patients from all groups in our study, regardless of FVC% predicted at baseline. Our data agree with previous publications and suggest that the disease progresses as fast in patients with preserved lung function as in patients with less preserved lung function at baseline [5, 19, 28]. In this sense, both post-hoc analyses of clinical trials and real-world data reports demonstrate that patients with preserved lung function equally benefit from antifibrotic treatment, and therefore support treatment at early stages of the disease [5, 11, 19, 36]. Receiving antifibrotic treatment is associated with a reduction of disease progression and an increased median survival irrespective of FVC % predicted at baseline [5, 11, 19, 36]. From a patient perspective, reducing disease progression as early as possible is important, given the irreversible nature of IPF and the inability of current treatment to improve symptoms once the disease has progressed [2]. In this line, an analysis of the population of the INMARK study highlights the importance of early treatment, since a 12-week delay in initiation of nintedanib seemed not to be fully compensated during a 52-week period [12].

Occurrence of AE-IPF is associated to a rapid patient FVC decline and higher mortality. Suffering at least one acute exacerbation during one year is associated with a higher risk of future mortality [4, 8], and preventing AE-IPF is one of the main goals of IPF treatment. In our study, patients with lower FVC reported more AE-IPF than patients with FVC > 50%; however, exacerbations also occurred in patients with well-preserved FVC, and suffering one exacerbation was linked with a clinically relevant decline in FVC. In this line, controlled clinical trials have reported a rate of 2.8% of exacerbations among patients with FVC > 90% during 12 months [11]. Regarding AE-IPF management, around 32% of AE-IPF events included corticosteroid, which is low compared with previously published evidence in which high-dose steroids use had been widely reported [37]. It may have been influenced by both, the severity and the definition of the AE-IPF. On the one hand, a lower severity may lead to a lower use of corticosteroid and lower doses. On the other hand, the AE-IPF definition used in this study was based on Collard et al. [24], which includes the appearance of new generalized alveolar abnormalities. This new broad definition could include secondary exacerbations such as infections, not included in previous definitions[38], which could have been managed with specific pharmacological treatments other than corticosteroids.

Early treatment of IPF with antifibrotics seems to be supported by data on FVC decline, reduction of AE-IPF events and improvement of survival. Moreover, recent studies suggest that treatment with antifibrotics may improve survival even when no differences in FVC decline can be detected [5, 19]. Nevertheless, a recent international survey that aimed to understand treatment patterns of IPF showed that half of the participating physicians would not treat IPF patients with “mild” or “stable” disease and was concerned about adverse effects of antifibrotic therapy [39]. Adverse events of antifibrotics are the most common reason for treatment discontinuation [35, 40]: however, in most patients they can be managed without dose reduction nor drug discontinuation [41]. The ‘wait and watch’ strategy seems to be still a common approach, despite it may jeopardize quality of life and survival of IPF patients [2, 39]. Factors explaining this low prescription pattern may include little knowledge about the risk/benefit of antifibrotic treatment, as well as restrictions due to public policies, among others [2, 39]. In this sense, improving education on approved antifibrotic therapy may help to change the trend towards early treatment to maintain patients' lung function and, consequently, may contribute to a decrease in the use of healthcare and non-healthcare resources associated with IPF.

Real-world data on IPF management is still scarce but is essential to understand current clinical practice and design effective therapeutic strategies. The prospective non-interventional design of this study allowed us to obtain detailed data on management and healthcare resources use for patients with confirmed IPF.

### Limitations

As all prospective cohort studies, the present study has some limitations related to its design, such as potential selection and recruitment bias that may limit its population representativeness. Moreover, clinical impairment of IPF patients during follow up may have impacted the availability of data for specific variables (e.g. inability to perform respiratory function tests). Estimation of use of some resources may have been affected by recall bias by patients and/or incomplete medical records. Of note, comorbidities that could impact resource utilization might not be fully captured in this study, since only treatments and resources related to IPF were collected. Finally, this study was carried out in Spain, and therefore, the results may not be valid for extrapolation to other countries.

## Conclusions

This study provides detailed information about real-world data on IPF management and associated resource use in Spain. The results show a significantly higher annual IPF-related resource use in patients with more impaired lung function at baseline compared with those with better preserved lung function.

During the study, FVC decline was observed irrespective of FVC% predicted at baseline. Therefore, slowing IPF progression to maintain patients at early disease stages would improve resource use and IPF clinical management.

## Supplementary Information

**Additional file 1: Table S1.** Non-health resources used along the study. **Table S2.** Non-health resources used during an AE-IPF.

## Data Availability

The datasets generated during and/or analyzed during the current study are not publicly available due to participants privacy protection but are available from the corresponding author on reasonable request.

## Abbreviations

6MWT: 6-Minute walk test
AE-IPF: Acute exacerbations of idiopathic pulmonary fibrosis
ANOVA: Analysis of variance
BMI: Body mass index
CI: Confidence interval
DL_CO_-c: Carbon monoxide lung diffusion capacity (corrected for haemoglobin)
EB: Ethical board
eCRF: Electronic case report form
FVC: Forced vital capacity
ICU: Intensive care unit
ILD: Interstitial lung disease
IPF: Idiopathic pulmonary fibrosis
NHS: National health system
NIMV: Non-invasive mechanical ventilation
SD: Standard deviation
SEPAR: Spanish Respiratory Society (Sociedad Española de Neumología y Cirugía Torácica)
US: United States
