# Supplementary material for: Clinical management and acute exacerbations in patients with idiopathic pulmonary fibrosis in Spain: results from the OASIS study
Source: Respir Res. 2022 Sep 7;23:235. doi: 10.1186/s12931-022-02154-y (PMC9450456; doi:10.1186/s12931-022-02154-y)
Supplement: Supplementary file 1 — Additional file 1: Table S1. Non-health resources used along the study. Table S2. Non-health resources used during an AE-IPF. [file 12931_2022_2154_MOESM1_ESM.docx]

**Additional tables**

**Table S1.** Non-health resources used along the study.

|  | **Total**  N=191 | **FVC<50%**  N=19 | **FVC 50-80%**  N=144 | **FVC>80%**  N=28 | **P value** |
| --- | --- | --- | --- | --- | --- |
| **Transport use**, n (%) | 21 (11.0%) | 7 (36.8%) | 13 (9.0%) | 1 (3.6%) | **0.0021** |
| Ambulance**^a^** | 19 (90.5%) | 6 (85.7%) | 12 (92.3%) | 1 (100.0%) | 1.0000 |
| Taxi**^a^** | 3 (14.3%) | 2 (28.6%) | 1 (7.7%) | 0 (0.0%) | 0.3744 |
|  | **Total**  N=166 | **FVC<50%**  N=14 | **FVC 50-80%**  N=130 | **FVC>80%**  N=22 | **P value** |
| **Use of orthoprosthetic material due to IPF** | 6 (3.6%) | 2 (14.3%) | 3 (2.3%) | 1 (4.5%) | 0.0732 |
| **Required structural changes at home^b^** | 2 (1.2%) | 0 (0.0%) | 1 (0.8%) | 1 (4.5%) | 0.3877 |
|  | **Total**  N=183 | **FVC<50%**  N=15 | **FVC 50-80%**  N=141 | **FVC>80%**  N=27 | **P value** |
| **Help from a caregiver** ^c^ | 41 (22.4%) | 6 (40.0%) | 31 (22.0%) | 4 (14.8%) | 0.4565 |
| Formal caregiver | 3 (7.3%) | 2 (33.3%) | 1 (3.2%) | 0 (0.0%) | 0.0723 |
| Informal caregiver | 39 (95.1%) | 5 (83.3%) | 30 (96.8%) | 4 (100.0%) | 0.4329 |
| **Time dedicated to patient care with IPF (hours/week, 7 days)**, mean (SD) |  |  |  |  |  |
| Formal caregiver**^a^** | 25.33 (34.39) | 36.00 (41.01) | 4.00 (.) | - | 0.5403 |
| Informal caregiver**^a^** | 45.05 (51.48) | 53.60 (36.66) | 43.23 (55.35) | 48.00 (44.65) | 0.6288 |

^a^ In case of affirmative response of previous item.

^b^ Structural change of the home (extension of doors, elevators, railings, anti-slip floors, electric chairs for stairs, more accessible bedroom...), elevators in the stairs in common areas, complementary technical aids (handles for the shower, seat for the bathtub, etc.), vehicle adaptation.

^c^ If the patient needed any help from a caregiver in the last 6 months (since the last visit). A same patient could require more than 1 type of caregiver.

**In bold, p-values <0.05.**

**Table S2.** Non-health resources used during an AE-IPF.

|  | **Total**  N=28 | **FVC<50%**  N=9 | **FVC 50-80%**  N=16 | **FVC>80%**  N=3 | **P value** |
| --- | --- | --- | --- | --- | --- |
| **Transport use**, n (%) | 12 (42.9%) | 4 (44.4%) | 8 (50.0%) | 0 (0.0%) | 0.4032 |
| Ambulance**^a^** | 12 (100.0%) | 4 (100.0%) | 8 (100.0%) | - |  |
| Taxi**^a^** | 0 (0.0%) | 0 (0.0%) | 0 (0.0%) | 0 (0.0%) |  |
| **Use of orthoprosthetic material due to IPF** | 1 (3.6%) | 1 (11.1%) | 0 (0.0%) | 0 (0.0%) | 0.4286 |
| **Required structural changes at home^b^** | 1 (3.6%) | 1 (11.1%) | 0 (0.0%) | 0 (0.0%) | 0.4286 |
| **Help from a caregiver** | 0 (0.0%) | 0 (0.0%) | 0 (0.0%) | 0 (0.0%) |  |
| **Economic aid and formal services** | 0 (0.0%) | 0 (0.0%) | 0 (0.0%) | 0 (0.0%) |  |

^a^ In case of affirmative response of the transport use requirement.

**^b^**Structural change of the home (extension of doors, elevators, railings, anti-slip floors, electric chairs for stairs, more accessible bedroom...), elevators in the stairs in common areas, complementary technical aids (handles for the shower, seat for the bathtub, etc.), vehicle adaptation.
